# The presence of bacteria within tissue provides insights into the pathogenesis of oral lichen planus

**DOI:** 10.1038/srep29186

**Published:** 2016-07-07

**Authors:** Yun Sik Choi, Yunji Kim, Hye-Jung Yoon, Keum Jin Baek, Jehan Alam, Hee Kyung Park, Youngnim Choi

**Affiliations:** 1 Departments of Oral Microbiology and Immunology, School of Dentistry and Dental Research Institute, Seoul National University, 101 Daehak-ro, Jongno-gu, Seoul 03080, Republic of Korea; 2 Oral Pathology, School of Dentistry and Dental Research Institute, Seoul National University, 101 Daehak-ro, Jongno-gu, Seoul 03080, Republic of Korea; 3 Oral Medicine and Oral Diagnosis, School of Dentistry and Dental Research Institute, Seoul National University, 101 Daehak-ro, Jongno-gu, Seoul 03080, Republic of Korea

## Abstract

Oral lichen planus (OLP) is a chronic T cell-mediated mucocutaneous disease of unknown etiopathogenesis. Although various antigens have been considered, what actually triggers the inflammatory response of T cells is unknown. In the present study, we propose that intracellular bacteria present within tissues trigger T cell infiltration and provide target antigens. Sections of OLP (n = 36) and normal (n = 10) oral mucosal tissues were subjected to *in situ* hybridization using a universal probe targeting the bacterial 16S rRNA gene and immunohistochemistry with anti-CD3, anti-CD4, anti-CD8, and anti-macrophage-specific antibodies. Bacteria were abundant throughout the epithelium and the lamina propria of OLP tissues, which exhibited positive correlations with the levels of infiltrated CD3^+^, CD4^+^, and CD8^+^ cells. Furthermore, bacteria were detected within the infiltrated T cells. Pyrosequencing analysis of the mucosal microbiota from OLP patients (n = 13) and control subjects (n = 11) revealed a decrease in *Streptococcus* and increases in gingivitis/periodontitis-associated bacteria in OLP lesions. Using the selected bacterial species, we demonstrated that certain oral bacteria damage the epithelial physical barrier, are internalized into epithelial cells or T cells, and induce production of T cell chemokines CXCL10 and CCL5. Our findings provide insights into the pathogenesis of OLP.

Oral lichen planus (OLP) is a chronic T-cell mediated mucocutaneous disease of unknown etiology^1^. OLP presents as papules, plaques, white striations, or erosive/ulcerative lesions typically bilaterally on the buccal mucosa, gingiva, and tongue^1^. The histopathological features of OLP include liquefaction of the basal layer of epithelia, band-like lymphocytic infiltration at the interface between the epithelia and submucosa, and degenerating keratinocytes^2^. The infiltrated lymphocytes are mainly CD4^+^ and CD8^+^ T cells, and CD8^+^ T cells are thought to mediate the degeneration/destruction of epithelial cells^1^.

Various intrinsic or extrinsic antigens have been speculated to trigger the inflammatory responses of T cells^1^,^3^. When a distinct etiology is identified to establish a cause-effect relationship for the lesions that are clinically and histologically similar to OLP, they are preferentially referred to as oral lichenoid lesions (OLL)^3^,^4^. OLL includes oral lichenoid contact lesions, oral lichenoid drug reactions, and oral lichenoid lesions of graft-versus-host disease^5^. Differential diagnosis of oral lichenoid drug reactions from OLP is often impractical because the withdrawal of the putative drug is potentially dangerous^1^. Although several histologic features are associated with OLL, OLL cannot be exclusively distinguished from OLP by histology^6^,^7^. Viral infections, expression of heat shock proteins, and stress have been suggested as possible etiological factors of OLP, but the etiopathogenesis of OLP remains unclear^1^,^3^. It has been proposed that the bacteria present within the gingival tissues drive the infiltration of inflammatory cells to the lesions of periodontitis, a chronic inflammation of the periodontium^8^,^9^. Abnormal features of OLP epithelium, such as atrophy, hyperkeratosis, acanthosis, and liquefaction of the basal layer^2^, suggest barrier dysfunction. We postulated that bacterial invasion into the mucosal tissue may be the cause of the immune cell infiltration observed in OLP lesions.

The surface of human body is colonized with microbiomes that coevolved with the host. Changes in human microbiota, which lead to an imbalance between protective and harmful bacteria, are associated with diverse localized or systemic diseases^10^. Periodontitis is a major oral disease caused by dysbiosis of subgingival microbiota^10^,^11^. Similarly, changes in the microbiota of the oral mucosa may be associated with OLP. However, little is known about the characteristics of oral microbiota in OLP.

In the present study, we report the presence of bacteria within the lamina propria and infiltrated T cells as well as the epithelium, which exhibited positive correlations with the levels of T cell infiltration in OLP tissues. Pyrosequencing analysis revealed changes in the mucosal microbiota associated with OLP. Using the selected bacterial species, we demonstrate that certain oral bacteria can damage the epithelial physical barrier, can be internalized into epithelial cells or T cells, and can induce production of T cell chemokines. These findings provide novel insights into the pathogenesis of OLP.

## Results

### Study population

For the present study, the mucosal bacterial samples and biopsies were obtained from 13 new patients (age 56.8 ± 3.3 years) diagnosed with OLP in the Oral Medicine Clinic, Seoul National University Dental Hospital (SNUDH). Six cases were diagnosed with OLP by both pathologists (OLP/OLP). Seven cases diagnosed with OLL by one or two pathologists (OLL/OLP) were included because the cases were clinically OLP, OLL cannot be differentiated from OLP by histology, and the OLL/OLP cases did not differ from the OLP/OLP cases in all clinical aspects, including treatment regimen, response to treatment, and lack of a cure for the disease. Detailed clinical information of the 13 patients is presented in Table 1. Mucosal bacterial samples were also obtained from 11 control subjects (age 52.5 ± 3.7 years) without oral mucosal disorders.

Additional tissue sections of 23 OLP cases without ulceration and 10 normal oral mucosa were obtained from the tissue bank at the Department of Oral Pathology, SNUDH. In total, 36 OLP cases were reviewed again by an oral pathologist and scored for each histopathologic feature of OLP and OLL^6^, as presented in Supplementary Table S1.

### Increased bacterial invasion into the lamina propria in OLP tissues

**Figure 1:**
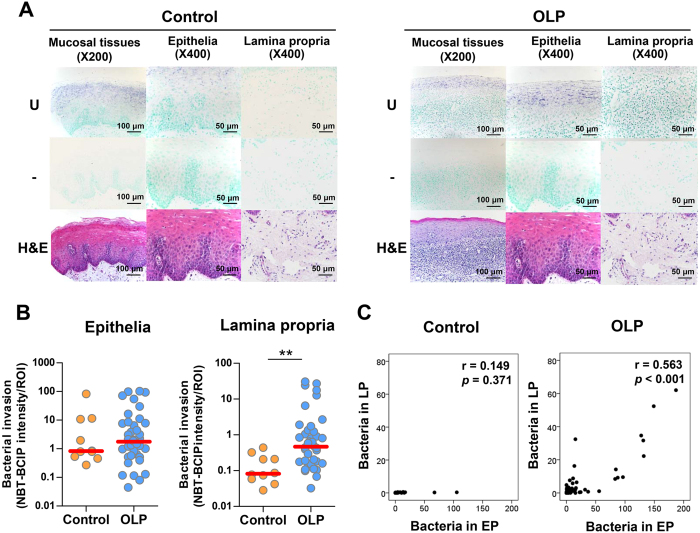
Increased bacterial invasion into the lamina propria in OLP tissues. (**A**) Representative *in situ* detection of bacteria in the control (n = 10) and OLP tissues (n = 36). U, a universal probe targeting bacterial 16S rRNA; -, a negative control probe mixed with 10-fold excess of unlabeled probe; H&E, hematoxylin and eosin stain. (**B**) The mean intensity of bacterial signals per region of interest (ROI) in the epithelia and lamina propria was analyzed using ImageJ software. The red bar indicates the median of each group (**P < 0.01 by Mann-Whitney U test). (**C**) Correlation plots between the levels of bacteria in the epithelia and those in the lamina propria in each group (*r* and *P* by Spearman’s rank correlation test).

To determine the presence of bacteria in OLP tissues, *in situ* hybridization was performed using a universal probe targeting bacterial 16S rRNA. In the control oral mucosa, the bacterial signals were often detected within the epithelia but rarely in the lamina propria. However, bacterial signals were detected in the lamina propria of all OLP tissues (Fig. 1A). Accordingly, the intensity of the bacterial signals in the lamina propria was increased in the OLP compared with control tissues, whereas that in the epithelia was not different (Fig. 1B). Although the total amount of bacteria detected within the epithelia was not different from the amount in control tissues, the incidence of bacterial detection within the basal layer of the epithelia was significantly different (10.0% vs. 72.5%), indicating this parameter as a risk factor for OLP (odds ratio [OR] 23.7, 95% confidence interval [95% CI] 2.7–209.8, *P* = 0.004). Additionally, the levels of bacteria detected within the lamina propria exhibited a significant positive correlation with those within the epithelia in the OLP (*r* = 0.563, *P* < 0.001), but not in the control tissues (*r* = 0.149, *P* = 0.371, Fig. 1C). Collectively, these results indicate that bacterial invasion into the lamina propria is increased in OLP tissues, which is consistent with liquefaction of the basal cell layer.

### Bacteria detected within T cells

**Figure 2:**
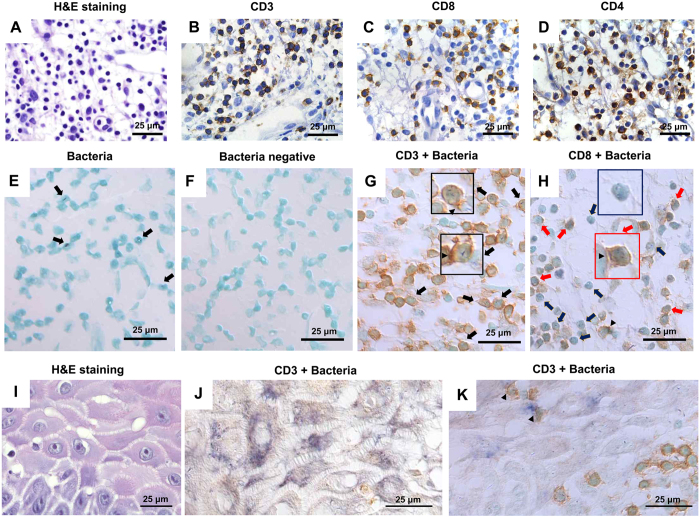
Presence of bacteria within epithelial cells CD3^+^, CD8^+^ and CD8^−^ lymphocytes in OLP tissues. Sections of OLP tissues were stained with hematoxylin and eosin (**A,I**) or immunostained for CD3 (**B**), CD8 (**C**), and CD4 (**D**). Sections of OLP tissues were hybridized *in situ* for 16S rRNA (**E**). Arrows indicate bacteria detected within lymphocytes. Sections of OLP tissues were hybridized *in situ* with a negative probe and then immunostained with an isotype antibody (**F**). Sections of OLP tissues (n = 4) were hybridized *in situ* for 16S rRNA and then immunostained for CD3 (**G,J,K**) or CD8 (**H**). Black arrows indicate bacteria signals detected within CD3^+^ cells; red arrows indicate bacteria detected within CD8^+^ cells; blue arrows indicate bacteria detected within CD8^−^ lymphocytes; arrowheads indicate an immunologic synapse-like structure.

All OLP tissues presented a band-like infiltration of lymphocytes that consisted primarily of T cells as shown by immunohistochemical staining of CD3, CD4, or CD8 (Fig. 2A–D). Interestingly, bacterial signals were often found to overlap with the nuclei of inflammatory cells of lymphocyte morphology (Fig. 2E). Bacterial invasion of human and murine T cells has been reported^12^,^13^,^14^. Therefore, dual detection of bacterial signals and either CD3 or CD8 was performed to determine if the infiltrated T cells in OLP tissues harbored bacteria. Differential interference contrast (DIC) microscopy revealed that almost all bacterial signals were confined to cellular structures rather than intercellular spaces in the lamina propria. Bacteria were observed within the cell boundaries of T cells labeled with CD3 or CD8 that appeared to be embedded in cells. Although dual detection of bacterial signals and CD4 was not performed due to non-specific staining of CD4 by dual staining, the bacterial signals were also observed within CD8^−^ lymphocytes, presumably CD4^+^ T cells (Fig. 2F–H). These results suggest that particular oral bacteria can cause intracellular infection of T cells in OLP tissues, representing the first demonstration of this phenomenon in human tissues. DIC microscopy further revealed that bacterial signals in the epithelia were also confined to the cellular structures of epithelial cells, some of which had contacts with infiltrated T cells (Fig. 2I–K). Interestingly, concentrated CD3 or CD8 was often observed at the contact sites of T cells with other cells that include epithelial cells, T cells, and an unidentified cell type, suggesting the formation of an immunologic synapse (arrowheads in Fig. 2G,H,K).

### Correlations between the amount of bacteria in the lamina propria and the infiltration of T cells

**Figure 3:**
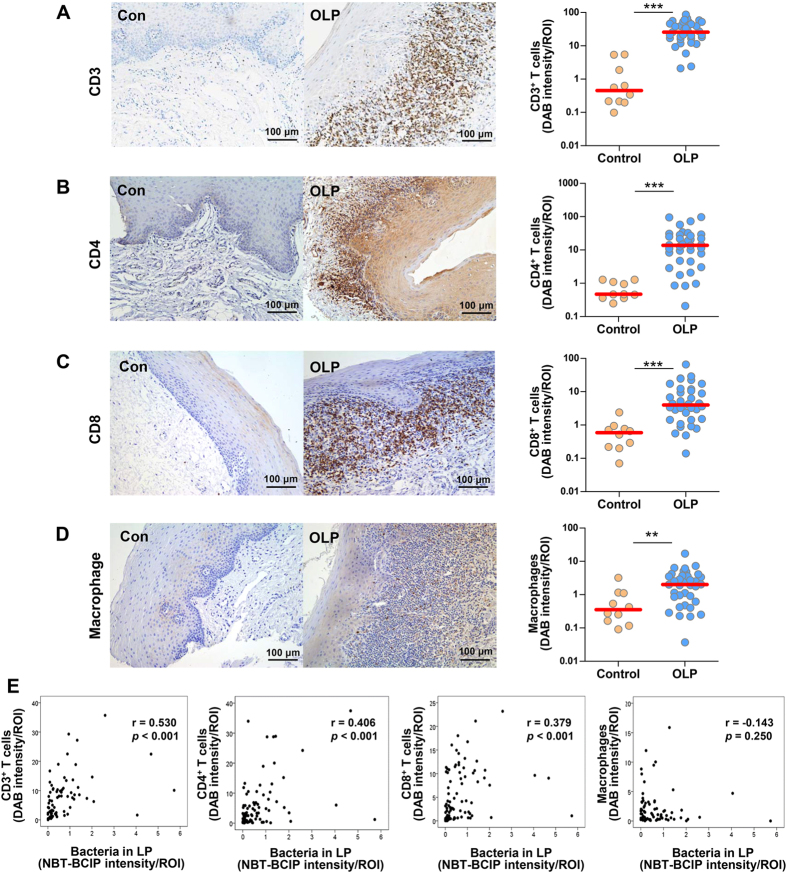
Increased infiltration of T cells correlated with the levels of bacteria in the lamina propria in OLP. (**A–D**) Representative immunohistochemical detection of CD3^+^ cells (**A**), CD4^+^ cells (**B**), CD8^+^ cells (**C**), and macrophages (**D**) in control (n = 10) and OLP (n = 36) tissues. Each symbol in the graphs represents the mean intensity of stained signals per ROI measured in four randomly chosen fields (**P < 0.01; ***P < 0.001 by Mann-Whitney U test). (**D**) Correlation plots between the levels of bacteria in the lamina propria and the levels of CD3^+^ cells, CD4^+^ cells, CD8^+^ cells, or macrophages (*r* and *P* by Spearman’s rank correlation test).

To examine the relationship between bacterial invasion of tissues and immune cell infiltration, the levels of CD3^+^, CD4^+^, and CD8^+^ cells and macrophages were determined by immunohistochemistry and quantitation. All cell types were increased in the OLP compared with the control tissues (Fig. 3A–D). However, only the levels of CD3^+^, CD4^+^ and CD8^+^ cells, i.e., not macrophages, exhibited significant positive correlations with the levels of bacteria detected within the lamina propria (Fig. 3E and Supplementary Fig. S1). These results suggest that bacterial invasion into the lamina propria is associated with T cell infiltration in OLP tissues.

### Changes in the oral mucosal microbiota associated with OLP

**Figure 4:**
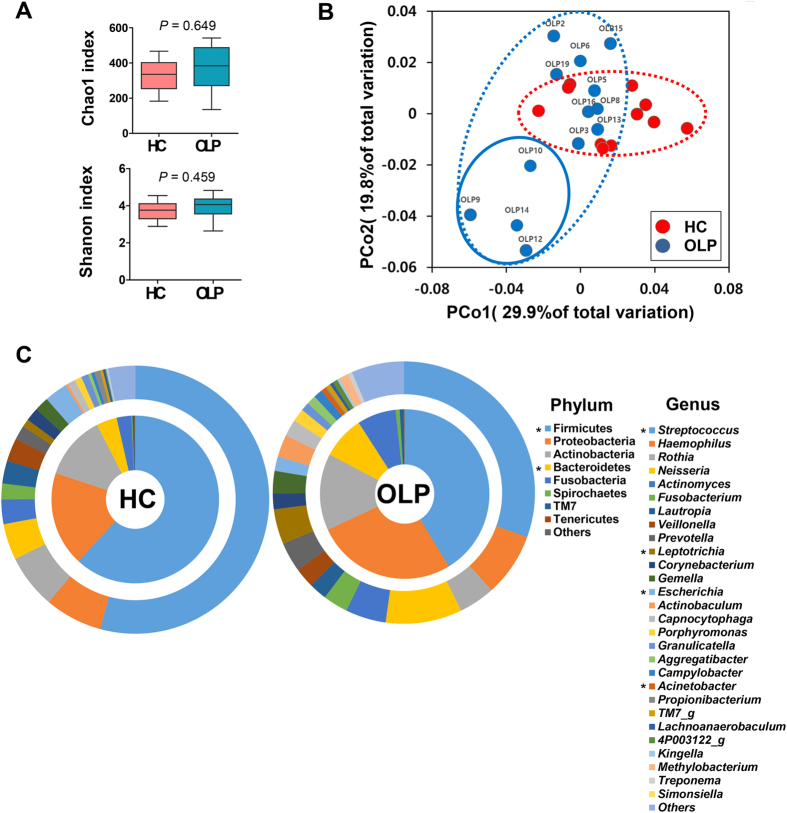
Altered mucosal microbiota in OLP. Bacterial communities of oral mucosa samples from healthy subjects (n = 11) and the lesion of OLP patients (n = 13) were analyzed by pyrosequencing of 16S rRNA gene. (**A**) The species richness and microbial diversity estimated by the Chao1 and Shannon diversity index, respectively, are expressed using box and whisker plots (*P* by Mann-Whitney U test). (**B**) PCoA plot generated using weighted Unifrac metric. (**C**) Double pie charts present the mean relative abundance of dominant phyla (>0.1% in control) and genera (>0.1% in control) (*P < 0.05 by Mann-Whitney U test).

The bacteria detected within the lamina propria of OLP tissues may be increased on the surface of oral mucosa. Therefore, the bacterial communities collected from the mucosal surface of OLP lesions were compared with those of healthy control subjects. The species richness determined by the Chao1 index (329 ± 26 vs. 369 ± 37, *P* = 0.649) and the microbial diversity determined by the Shannon index (3.72 ± 0.15 vs. 3.98 ± 0.16, *P* = 0.459) were not significantly different between the two groups (Fig. 4A). In the principal coordinates analysis (PCoA) plot, the control and OLP samples clustered separately with some ovrelap (Fig. 4B). Comparison of the relative abundance of each taxon revealed many differences in the composition of the microbiota between the control and OLP samples. At the phylum level, the relative abundance of Firmicutes was significantly decreased in OLP, but the abundance of Bacteroidetes was increased. At the genus level, the abundances of *Streptococcus* and *Escherichia* were decreased in OLP mucosa, whereas the abundances of *Leptotrichia* and *Acinetobacter* were increased (Fig. 4C). Among the 31 species/phylotypes that showed significant differences in relative abundance between the two groups, only six species/phylotypes were decreased in the OLP group compared with the controls, whereas 25 species/phylotypes were increased (Table 2). Specifically, a decrease in the relative abundance (per 0.01%) of *Streptococcaceae_uc_s* was associated with OLP risk (OR 0.405, CI 95% 0.186–0.883, *P* = 0.023). The model also revealed an association between OLP risk and an increase (per %) in *Capnocytophaga gingivalis*, although the statistical significance was not reached (OR 2.0E12, CI 95% 0.082–6.0E25, *P* = 0.072).

We further analyzed the microbiota of four OLP samples (circled with blue solid line in Fig. 4B) that presented complete separation from the control samples in the PCoA plot. Fourteen of the 31 species/phylotypes listed in Table 2 maintained significant differences from the control samples. Decreases in five species/phylotypes and increases in 26 species/phylotypes compared to the control group were additionally observed (Supplementary Table S2). However, none of the clinical or histologic parameters was significantly associated with the OLP subset with distinct microbiota.

### Internalization of selected bacterial species into human oral epithelial cells and leukocytes

**Figure 5:**
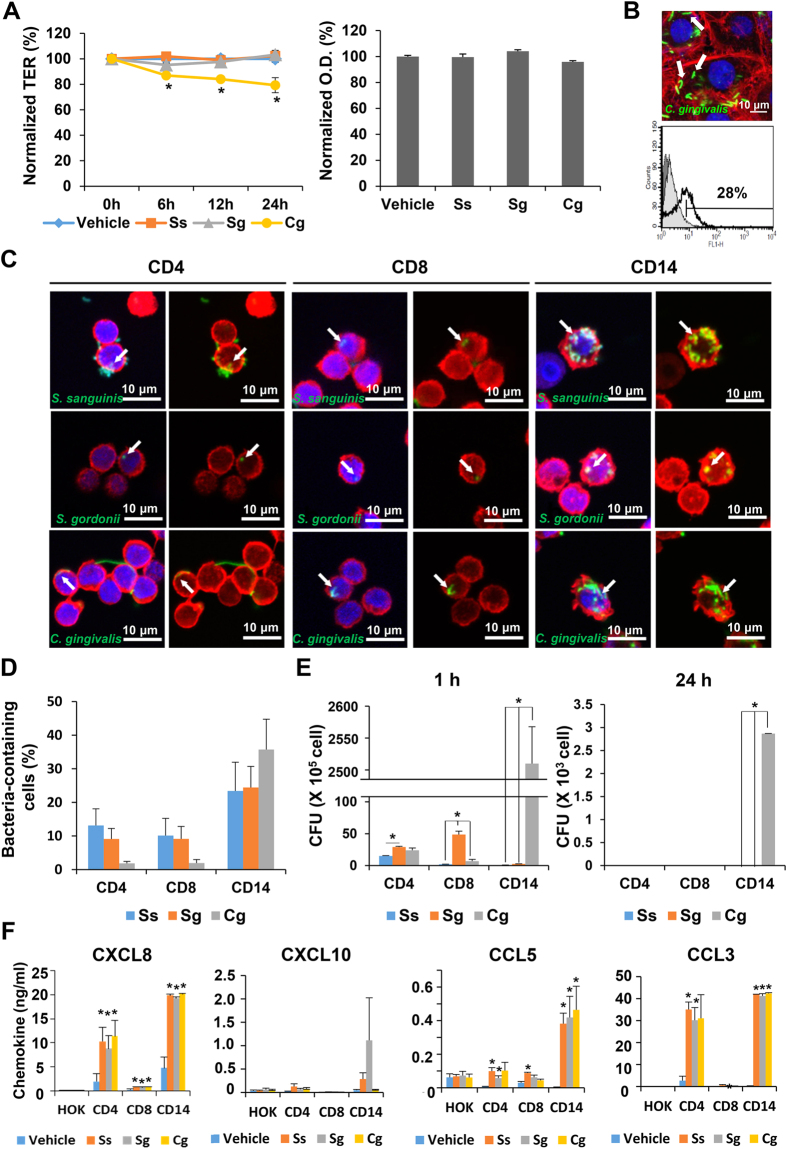
Internalization of selected oral bacterial species into host cells and chemokine induction. (**A**) Tight-junctioned monolayers of HOK-16B cells were infected with *S. sanguines* (Ss), *S. gordonii* (Sg), and *C. gingivalis* (Cg) at an MOI of 500. Left: TER measurement at 0, 6, 12, and 24 hours expressed as the relative percentage compared with baseline (s.e.m., n = 6, *P < 0.05 by t-test). Right: cell viability measured using a CCK-8 assay kit and expressed as a relative percentage of vehicle control (s.e.m., n = 6). (**B**) HOK-16B cells were infected with CFSE-labeled bacteria at MOI 1000 for 24 h. Top: confocal microscopy of CFSE-labeled *C. gingivalis* (green) internalized into HOK-16B cells. White arrows indicate bacteria within the cell boundary. Blue, Hoechst 33342; red, rhodamine phalloidin. Scale bar, 10 μm. Bottom: HOK-16B cells containing internalized CFSE-labeled *C. gingivalis* (thick empty line) analyzed by flow cytometry were overlaid over negative controls (dark gray, fixed cells infected with bacteria; light gray, non-infected live cells). (**C,D**) Purified human CD4^+^, CD8^+^, or CD14^+^ cells were infected with CFSE-labeled bacteria at MOI 1000 for 1 hour (n = 3 different donors). (**C**) Representative confocal microscopy of CFSE-labeled bacteria (green) internalized into human CD4^+^, CD8^+^, or CD14^+^ cells. White arrows indicate bacteria within the cell boundary. Blue, Hoechst 33342; red, rhodamine phalloidin. (**D**) The percentage of cells containing the internalized bacteria analyzed by flow cytometry (s.e.m.). (**E**) Purified human CD4^+^, CD8^+^, or CD14^+^ cells were infected with bacteria at MOI 1000 for 1 hour and further cultured for 1 and 24 hours in the presence of gentamicin. After lysing the cells, bacteria in the lysates were cultured on blood agar plates (s.e.m., n = 2 different donors, *P < 0.05 by one-way ANOVA with Tukey’s post hoc). (**F**) The amounts of CXCL8, CXCL10, CCL5, and CCL3 in the culture supernatant of HOK-16B, CD4^+^, CD8^+^, or CD14^+^ cells infected with bacteria at MOI 1000 for 1 hour and further cultured for 24 hours with gentamicin (s.e.m., n = 3 different donors, *P < 0.05 by t-test).

Many cocci and some fusiform bacteria were observed in OLP tissues by *in situ* hybridization, and domination of *Streptococci* over the diverse flora within buccal cells has been reported^15^. Therefore, *C. gingivalis* and two *Streptococci* species, *S. sanguinis* and *S. gordonii*, were selected for additional experiments to investigate the potential roles of bacteria in the etiopathogenesis of OLP. Diverse pathogens can modulate the physical barrier function of epithelia to facilitate infection^16^,^17^,^18^. Therefore, the effect of bacteria on physical barrier function was examined using trans-epithelial electrical resistance (TER) of immortalized human oral keratinocyte (HOK)-16B cells as a readout. Only *C. gingivalis* induced a significant decrease in TER in a time-dependent manner without affecting the viability of HOK-16B cells (Fig. 5A). *S. gordonii*, but not *S. sanguinis*, is known to invade into HOK-16B cells^19^. The internalization of *C. gingivalis* into HOK-16B cells was examined using bacteria labeled with 5-(and 6-) carboxy-fluorescein diacetate succinimidyl ester (CFSE). *C. gingivalis* was clearly observed within the cell boundary by confocal microscopy, and flow cytometric analysis revealed that 28% of HOK-16B cells had internalized bacteria (Fig. 5B).

We also investigated whether the selected bacterial species can be internalized into T cells as observed in the OLP tissues. Surprisingly, all three species were detected within the cell boundary surrounded by actin filaments of CD3^+^, CD4^+^, and CD8^+^ cells, as well as within the CD14^+^ cells (Fig. 5C and Supplementary Fig. S2A). To exclude the possibility that the bacteria-containing cells in CD3^+^, CD4^+^, or CD8^+^ populations are a few contaminating monocytes, the infected cells were also analyzed by flow cytometry with gating on the lymphocytes (Supplementary Fig. S3). Although there was donor-to-donor variation, approximately 2–13% of CD3^+^, CD4^+^, or CD8^+^ cells had internalized bacteria for all three species (Fig. 5D and Supplementary Fig. S2B). The ability of the internalized bacteria to survive within host cells was examined using an antibiotic protection assay. One hour after infection, viable bacteria were isolated from the CD4^+^ or CD8^+^ cells for all three species, which did not survive 24 hours after infection. Interestingly, only *C. gingivalis* survived within the CD14^+^ cells, even after 24 hours of infection (Fig. 5E).

### Induction of chemokines from host cells by selected bacteria

Increased levels of chemokines, including CCL3/MIP-1α, CCL5/RANTES, CXCL8/IL-8, and CXCL10/IP-10, have been implicated in OLP^20^,^21^. To investigate the role of bacteria in the pathogenesis of OLP, the levels of these chemokines in the culture supernatant of HOK-16B, CD4^+^, CD8^+^, and CD14^+^ cells infected with the three bacterial species were measured. Upon bacterial challenge, CD14^+^ cells efficiently induced all four chemokines. CD4^+^ cells also produced CCL3 and CXCL8 at high levels and CCL5 and CXCL10 at substantial levels. CD8^+^ cells upregulated only CCL3 and CCL5 at low levels, and HOK-16B cells minimally responded to bacterial challenge. No differences in the ability to induce chemokines were observed among the three species (Fig. 5F). These results suggest that bacteria can efficiently induce chemokines from the cells in the lamina propria.

## Discussion

OLP is a chronic T-cell mediated inflammatory disease. However, the cause of the inflammatory response of T cells in OLP is unknown. In the present study, we report the presence of bacteria within epithelial cells and infiltrated T cells in OLP tissues, and we propose the intracellular bacteria as an important triggering factor of T cell infiltration.

The mucosal epithelia provide barriers against invading microbes. Although all OLP tissues were obtained from the area without ulceration, any bacteria that had invaded the tissue through ulcerated regions might have spread to non-ulcerated areas. However, bacteria were also detected in tissues from patients who had no ulceration and limited erosive lesion (Supplementary Fig. S4). Unlike the control tissues, in which bacterial invasion ceased within the prickle cell layer of epithelia, the epithelia in OLP tissues did not provide a barrier against invading bacteria, as noted by the abundant bacterial signals throughout the basal layer of epithelium and lamina propria. The significant positive correlation between the levels of bacteria in the epithelia and the lamina propria in OLP, but not in control tissues, supports the view that the barrier function is damaged in OLP tissues.

The mucosal microbiota of OLP was characterized by a substantial decrease in *Streptococcus*, which was the most abundant genus in the oral mucosa of healthy subjects, and increases in multiple minor genera and species/phylotypes. Among the 25 species/phylotypes increased in OLP tissues, nine species, including *Fusobacterium nucleatum, Neisseria oralis, C. gingivalis, Leptotrichia hongkongensis, Eikenella corrodens, T. denticola, T. socranskii, Centipeda periodontii*, and *Selenomonas sputigena*, are associated with gingivitis or periodontitis^22^,^23^,^24^. The decreases in *Streptococcal* species were more evident in the small subset of OLP group that was completely separated from the control group in the PCoA plot. When differences in the mucosal microbiota between the OLL/OLP and OLP/OLP cases were analyzed, the two groups had similar distribution in the PCoA plot (Supplementary Fig. S5). Most of the 31 species/phylotypes that showed differences in relative abundance between the control and OLP groups also showed differences between the control group and the OLL/OLP and OLP/OLP groups separately (Supplementary Table S3), indicating minor differences in the composition of mucosal microbiota between the OLL/OLP and OLP/OLP groups.

The periodontal status of the control and OLP subjects was not evaluated in the current study, and the increase in gingivitis/periodontitis-associated bacteria may reflect the poor periodontal health of OLP patients. According to the literature, OLP patients have relatively worse periodontal health than controls, regardless of the extent of the lesions or clinical presentation^25^. Two groups have reported that plaque control and periodontal treatment improved the clinical features and painful symptoms of OLP with gingival involvement^26^,^27^. These findings suggest that the periodontitis-associated bacteria may also have a role in the pathogenesis of OLP.

Notably, *C. gingivalis* decreased TER and was directly internalized into oral epithelial cells *in vitro*. Among the bacterial species increased in the OLP mucosa, a periodontal pathogen *T. denticola* damages the epithelial physical barriers, which involves bacterial proteases^28^. *C. gingivalis* has a trypsin-like protease^29^ that may degrade the junctional proteins. Therefore, the bacterial invasion of mucosal tissues observed in OLP may be associated with the changes in the mucosal microbiota as well as with degeneration/atrophy of epithelial cells. Additionally, *F. nucleatum, E. corrodens*, and *T. denticola*, which can invade oral epithelial cells^15^,^19^, were increased in the OLP mucosa. Human oral keratinocytes express MHC class I molecules constitutively and HLA-DR upon stimulation with IFNγ or TNFα that are expressed by mononuclear cells adjacent to basal keratinocytes in OLP tissues^30^,^31^. Therefore, bacteria internalized into oral epithelial cells may be processed and presented to T cells, making their host cells the target of T cells^32^. Actually, immunologic synapse-like structures were often observed at the contact sites between the infected epithelial cells and T cells.

The most interesting finding in this study was the presence of bacteria within the infiltrated T cells in OLP tissues. The ability of T cells to uptake selected oral bacteria *in vitro* was confirmed by confocal microscopy, flow cytometry, and the antibiotic protection assay. The internalization of bacteria into T cells has been reported by only two research groups. Phalipon and colleagues reported that *Shigella flexneri* actively invades T cells by injecting IpgD through the type three secretion system and inhibits CD4^+^ T cell migration, impeding the priming of an effective protective response^12^,^13^. In contrast, Cruz-Adalia *et al*.^14^ reported that T cells can capture bacteria, such as *Listeria monocytogenes, Staphylococcus aureus*, and *Salmonella enterica* serovar *enteritidis*, by transinfection from dendritic cells and can efficiently kill the captured bacteria, conferring protection in mice. Although the mechanism by which oral bacteria are internalized into T cells and the sequelae of bacterial internalization are unclear, the abundant intracellular bacteria may be an important triggering factor for the T cell-dominant infiltration observed in OLP. Activated human T cells express MHC II molecules as well as MHC I and present the intracellular antigens to both CD4^+^ and CD8^+^ T cells^33^,^34^. Iijima *et al*.^20^ reported that infiltrating T cells in OLP tissues express not only CCR5 and CXCR3 but also their respective ligands CCL5 and CXCL10, suggesting a self-recruiting mechanism. Effector or memory T cells express CCL5 upon recognition of specific antigens^35^. As suggested by the immunologic synapse-like structures at the contact sites of T cells with other cells, a number of infiltrated T cells in OLP tissues may be specific to the intracellular bacterial antigens and produce CCL5 upon antigen recognition.

Known as a chemoattractant for neutrophils, CXCL8 can also recruit T cells, particularly activated or effector/memory T cell types^36^,^37^. The role of CXCL8 in the pathogenesis of OLP requires further study because there are conflicting reports regarding its expression in OLP tissues^21^,^38^. We postulate neutrophils are recruited when extracellular bacteria release n-formylated peptides, another potent neutrophil chemoattractant. However, such distinct extracellular bacteria were hardly detected in the OLP tissues.

The current study has several limitations. First, bacterial species observed within the OLP tissues was not clarified. Second, whether or not the infiltrated T cells are specific to the bacteria *in situ* is not known. Third, the altered mucosal microbiota observed in OLP patients do not provide evidence of a causal relationship. The shift in the mucosal microbiota could result from the altered mucosal surface initiated by unknown host factors. Danielsson *et al*. analyzed the transcriptomes of OLP and normal oral epithelia and reported that the differentially expressed genes involve epithelial differentiation and development, suggesting changes in the epithelial barrier in OLP tissues^39^. For example, the top 20 down-regulated genes in OLP include the MUC21 and CLDN7, which encode a cell-surface-associated Mucin-21 protein and a tight-junction protein claudin-7, respectively^39^.

**Figure 6:**
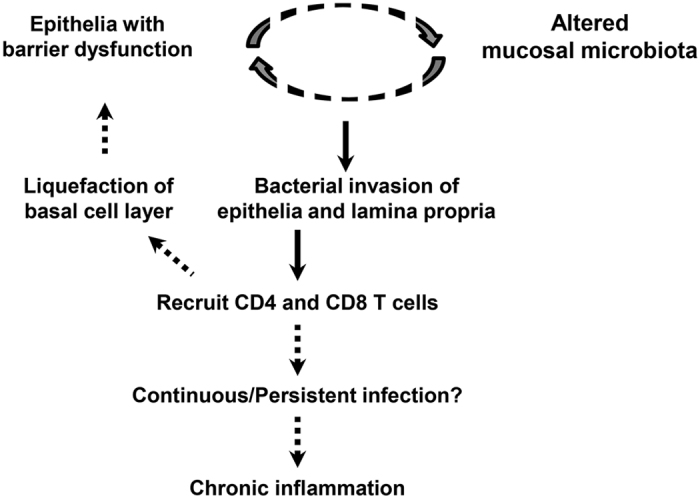
Proposed pathogenesis model for OLP. Solid and broken arrows indicate proved and unproved relations, respectively, in the current study.

Within these limitations, we propose a pathogenesis model for OLP based on the observed results (Fig. 6). The epithelia with barrier dysfunction and altered mucosal microbiota are likely to affect each other^40^, leading to the bacterial invasion of the epithelia and lamina propria. The intracellular bacteria may be presented to the infiltrated T cells as target antigens, resulting in liquefaction of the basal cell layer and further barrier dysfunction. This vicious cycle may cause continuous/persistent infection and chronic inflammation.

Collectively, our data implicate a novel role of bacteria in the pathogenesis of OLP in which bacteria damage the epithelial barriers, are internalized into epithelial cells and T cells, and induce T cell chemokines. This result suggests antibiotics as a new therapeutic option for OLP. We also demonstrated that *in situ* detection of bacteria combined with microbiota analysis provides a useful tool to study the potential role of bacteria in other diseases.

## Materials and Methods

Details of the methods are provided in the Supplementary materials and methods.

### Sample collection

All procedures involving human subjects and materials were performed in accordance with the Helsinki Declaration under approved protocols from the Institutional Review Boards at the SNUDH (CRI 12032 and CRI12023) and at Seoul National University, School of Dentistry (S-D20150007). Informed consent was obtained from all subjects. Subjects who had received antibiotics or steroid within the last month, patients with xerostomia (unstimulated whole salivary flow rate <0.1 ml/min), and smokers were excluded. Cases diagnosed with candidiasis or chronic mucositis by histopathology were also excluded. The exclusion criteria did not include systemic disease and medication. For the 13 newly diagnosed OLP patients, OLP lesions were objectively evaluated based on the number of sites with reticulation/keratosis, erythema, and ulceration, which correlates well with the pain of patients^41^. The subjective pain of patients was scored by the numerical rating scale^42^. Bacterial sampling and a punch biopsy were performed on the reticular lesion with or without erythema but with no ulceration located at the buccal mucosa of OLP patients. Bacterial samples were obtained from the buccal mucosa (n = 11) of control subjects (5 males and 6 females). For the bacterial sampling, subjects were asked to avoid eating and antiseptic mouthwashes for two hours before sampling, and a sterilized 20 × 10 mm polyvinylidene difluoride membrane was placed on the mucosa for 30 seconds.

### *In situ* hybridization, immunohistochemistry, and image analysis

*In situ* hybridization using a digoxigenin (DIG)-labeled universal probe targeting the 16S rRNA gene was performed as previously described^43^. Briefly, paraffin-embedded sections (4 μm) were subjected to de-paraffinization, re-hydration, and a sequential pre-treatment with 0.1 N HCl, 1 μg/ml proteinase K, and 0.1 M triethanolamine-HCl. The tissue sections were then hybridized with a DIG-labeled probe. As a negative control, hybridization was performed with the labeled probe with a 10-fold excess amount of non-labeled probe. The bound probe was detected with alkaline phosphatase-conjugated anti-DIG antibody and visualized with a nitroblue tetrazolium/5-bromo-4-chloro-3-indolyl phosphate (NBT-BCIP) solution. The sections were counter stained with methyl green.

For immunohistochemistry, antigens in the de-paraffinized and re-hydrated sections were retrieved by heating in citrate buffer. The tissue sections were incubated with anti-CD3 antibody clone CD3-12 (Serotec, Blackthorn, Bicester, England), anti-CD4 antibody clone 4B12 (Monosan, Sanbio, Uden, Netherlands), anti-CD8 antibody clone 4B11 (Serotec), or anti-macrophage-specific antibody clone 3A5 (Serotec). The bound primary antibodies were detected using a peroxidase-based detection kit. After a DAB reaction, the sections were counterstained with hematoxylin. For dual detection of bacteria and CD8, the tissue sections were first subjected to *in situ* hybridization of 16S rRNA followed by immunohistochemical detection of CD3 or CD8.

Four areas with various degrees of inflammatory infiltration were randomly chosen in the H&E-stained section of each sample. The same areas in the sections with *in situ* hybridization or immunohistochemistry were then photographed. The signals of *in situ* hybridization and immunohistochemistry were quantified using ImageJ software (National Institute of Mental Health, Bethesda, MD, USA).

### Pyrosequencing and data analysis

Genomic DNA was isolated from the mucosal samples using the Power Soil DNA Isolation Kit (MO BIO Laboratories, Carlsbad, CA, USA). The amplification and sequencing of 16S rRNA genes were performed at ChunLab, Inc. (Seoul, Korea) according to the previously described method using a 454 GS FLX Titanum Sequencing System (Roche Applied Science, Branford, CT, USA)^44^. The pyrosequencing data are available in the SRP database under the accession number SRP049562 and SRP065981.

The sequenced data were analyzed by using software provided by ChunLab, Inc. After removing PCR primer sequences, reads containing two or more ambiguous nucleotides and reads shorter than 300 bp were discarded. Chimera sequences detected by the Bellerophone method were also removed. The taxonomic classification of each read was assigned against the EzTaxon database-e (http://eztaxon-e.ezbiocloud.net). The cutoff values used for assigning each read to taxonomic assignment were as follows: species (*x* ≥ 97%), genus (97 > *x* ≥ 94%), family (94 > *x* ≥ 90%), order (90 > *x* ≥ 85%), class (85 > *x* ≥ 80%), and phylum (80 > *x* ≥ 75%), where *x* denotes similarity. The species richness and diversity index were calculated using the Ribosomal RNA database project’s pyrosequencing pipeline (http://pyro.cme.msu.edu). The cutoff value for assigning a sequence to the same group (phylotype) was equal to or greater than 97% similarity. Random subsampling was conducted to equalize variation in the read counts among the samples. The overall phylogenetic distance between communities was estimated using the weighted Fast UniFrac and was visualized using PCoA.

### Bacteria

*S. sanguinis* ATCC 804 (American Type Culture Collection, Manassas, VA, USA), *S. gordonii* ATCC 10558 (ATCC), and *C. gingivalis* KCOM 1581 (Korean Collection for Oral Microbiology, Gwangju, Korea) were used. For florescent application, bacteria were stained with CFSE (Molecular Probes, Eugene, OR, USA).

### Measurement of TER after bacterial infection

Immortalized human oral keratinocyte (HOK-16B) cells that originated from retromolar gingival tissues^45^ were plated onto a 3 μm-pore-size polycarbonate filter of a 24-well plate of the transwell two-chamber tissue culture system. The cells were cultured for 2 or 3 days until a confluent monolayer reached the peak resistance of approximately 15 Ω. Then, the cells were infected with *S. sanguines, S. gordonii*, and *C. gingivalis* at the multiplicity of infection (MOI) of 500. TER was measured at 0, 6, 12, and 24 hours using an ERS Volt-Ohm Meter (Millipore Bedford, MA, USA).

### Purification of primary human CD3^+^, CD4^+^, CD8^+^, and D14^+^ cells

Peripheral blood purchased from the Red Cross was used. Peripheral blood mononuclear cells isolated by the ficoll-hypaque method were sequentially subjected to purification of CD14^+^, CD4^+^, and CD8^+^ cells using magnetic beads conjugated with an appropriate antibody (BD Biosciences, San Diego, CA, USA), according to the manufacturer’s instructions. In separate experiments, CD3^+^ cells were purified using magnetic beads.

### Bacterial internalization into human cells

HOK-16B cells (3 × 10^4^ cells) were infected at 70% confluence with the CFSE-labeled bacteria at an MOI of 1,000 for 24 hours. Purified human CD3^+^, CD4^+^, CD8^+^, or CD14^+^ cells (2.5 × 10^5^ cells) were infected with the CFSE-labeled bacteria at an MOI of 1,000 for 1 hour. For confocal microscopic examination, the infected cells were fixed, permeabilized, and then stained with rhodamine-phallodin (Molecular Probes) and Hochest 33342 (Molecular Probes). Mounted slides were imaged using a Zeiss LSM700 confocal microscope (Carl Zeiss, Oberkochen, Germany) with serial z-sections. For flow cytometric analysis, the infected cells were washed with PBS and resuspended in trypan blue (400 mg/ml prepared in 0.85% saline solution) to quench the fluorescence of the extracellular bacteria. The cells were analyzed using a FACSCalibur (BD Biosciences). For the antibiotic protection assay, the infected cells were further cultured for 1 and 24 hours in the presence of gentamicin (50 μg/ml). The cells were lysed with sterile distilled water containing 0.5% saponin, and the lysates were plated onto a blood agar plate and cultured under an appropriate atmosphere for 2 to 3 days. The numbers of bacteria that survived within the cells are expressed as colony forming units (CFUs).

### Chemokine ELISA and multiplex assay

HOK-16B, CD4^+^, CD8^+^, and CD14^+^ cells were infected with three bacterial species for 1 hour and further cultured in the presence of gentamicin (50 μg/ml) for 24 hours. The culture supernatant were harvested and stored at −80 °C until use. The amount of CXCL8 in the culture supernatant was measured using an ELISA kit (R&D Systems, Minneapolis, MN, USA). The amounts of CCL3, CCL5, and CXCL10 were determined using a multiplex assay kit (R&D Systems).

### Statistical analyses

Non-parametric methods, including the Mann-Whitney U-test, Kruskal Wallis test, and Spearman’s rank correlation test were used to analyze the data from *ex vivo* experiments using tissue sections and bacterial samples. The t-test and ANOVA were used to analyze the data from *in vitro* experiments using HOK-16B cells and purified leukocytes. The association of bacterial species with OLP risk was determined with a logistic regression analysis. All statistical analyses were performed using SPSS Statistics 22 software (SPSS Inc., Chicago, IL, USA). Significance was set at *P* < 0.05.

## Additional Information

**How to cite this article**: Choi, Y. S. *et al*. The presence of bacteria within tissue provides insights into the pathogenesis of oral lichen planus. *Sci. Rep.* **6**, 29186; doi: 10.1038/srep29186 (2016).

## Supplementary Material

Supplementary Information

## Figures and Tables

**Table 1:** Clinical information of OLP patients.

No	Age	Sex	REU^a^ scoring		Pain score	Duration	Sites	Systemic disease with medication	Pathologist 1	Pathologist 2
			REU	Score						
OLP2	67	M	R1E2	4	2	1 year	Buccal mucosa	Hypertension	OLL	OLL
OLP3	59	F	R5E5U1	14.5	8	5 month	Buccal mucosa, Gingiva, Tongue	None	OLP	OLP
OLP5	69	F	R1E2U1	6	4	2 month	Buccal mucosa	Hypertension	OLP	OLL
OLP6	41	F	R2E2U2	9	5	6 month	Buccal mucosa	None	OLP	OLP
OLP8	65	F	R2E2U1	7	4	3 month	Buccal mucosa, Lip, Gingiva	None	OLL	OLL
OLP9	50	F	R3E8U2	19	5	6 month	Buccal mucosa, Gingiva	None	OLL	OLL
OLP10	29	M	R1E1	2.5	4	3 month	Buccal mucosa	Manic depression	OLP	OLL
OLP12	70	M	R1E2U1	6	5	6 month	Buccal mucosa	Hypertension, Diabetes	OLP	OLP
OLP13	53	M	R3E1	4.5	4	10 month	Buccal mucosa, Tongue, Palate	None	OLL	OLL
OLP14	56	M	R3E2U1	8	4	3 month	Buccal mucosa, Palate	Hypertension, Paresthesia	OLP	OLP
OLP15	56	M	R1E2	4	4	1 month	Buccal mucosa	None	OLP	OLP
OLP16	58	M	R4E9U4	25.5	7	4 year	Buccal mucosa, Gingiva, Tongue	Colon cancer	OLP	OLL
OLP19	66	F	R5E8	17	4	6 month	Buccal mucosa, Gingiva, Palate	None	OLP	OLP

^a^R, reticulation/keratosis; E, erythema; U, ulceration.

**Table 2:** Relative abundance (%)^a^ of species/phylotypes differently distributed between the control and OLP mucosal microbiota.

Species/phylotypes	Control (n = 11)	OLP (n = 13)	*P* value^c^
*HQ767899_s (Streptococcus*)	2.401 (0–42.911)	0.040 (6.524, 0)	0.030
*Escherichia coli group*	0.449 (13.088, 0.029)	0.066 (18.242, 0)	0.022
***Fusobacterium nucleatumd***^d^	0.191 (4.018, 0.007)	1.201 (6.187, 0.158)	0.036
***Neisseria oralisd***^d^	0 (0.156, 0)	0.107 (33.771, 0)	0.003
***Capnocytophaga gingivalis***^d^	0.089 (0.606, 0)	0.176 (2.511, 0)	0.036
***Leptotrichia hongkongensis***^d^	0.020 (0.748, 0)	0.139 (1.623, 0)	0.036
*Stomatobaculum longum*	0.025 (0.450, 0)	0.175 (1.257, 0)	0.028
*Aggregatibacter segnis*	0 (0.227, 0)	0.070 (2.570, 0)	0.022
*Actinomyces meyeri*	0.018 (0.106, 0)	0.161 (2.855, 0)	0.009
***Eikenella corrodens***	0.014 (0.216, 0)	0.056 (3.703, 0.007)	0.008
*4P004975_s (Actinomyces*)	0.039 (1.182, 0)	0 (1.119, 0)	0.016
*EF016847_s (EF016847_g)*	0.065 (1.124, 0.009)	0 (1.160, 0)	0.006
*Capnocytophaga sputigena*	0.025 (0.065, 0)	0.148 (0.874, 0)	0.001
*Leptotrichia buccalis*	0 (0.030, 0)	0.071 (3.624, 0)	0.013
*Prevotella oulorum*	0 (0.830, 0)	0.023 (0.780, 0)	0.036
*Streptococcus vestibularis*	0.033 (0.334, 0.008)	0 (0.060, 0)	0.001
*Megasphaera micronuciformis*	0 (0.238, 0)	0.057 (0.340, 0)	0.003
*AF385572_s (Leptotrichia*)	0 (0.036, 0)	0.040 (0.744, 0)	0.009
*AF385518_s (Leptotrichia*)	0 (0.039, 0)	0.034 (0.382, 0)	0.041
*Myxococcus virescens group*	0 (0, 0)	0 (0.608, 0)	0.028
*Mogibacterium vescum*	0 (0.053, 0)	0.028 (0.306, 0)	0.032
*Blautia wexlerae*	0 (0, 0)	0.010 (0.321, 0)	0.013
*AF385506_s* (TM 7_f)	0 (0.059, 0)	0.030 (0.198, 0)	0.028
***Treponema denticolad***^d^	0 (0.102, 0)	0.024 (0.144, 0)	0.011
***Treponema socranskiid***^d^	0 (0.053, 0)	0.027 (0.157, 0)	0.047
*AF385554_s (Prevotella*)	0 (0.40, 0)	0.014 (0.146, 0)	0.024
*AY134896_s (Leptotrichia*)	0 (0, 0)	0 (0.327, 0)	0.028
*Streptococcaceae_uc_s*	0.023 (0.059, 0.007)	0 (0.044, 0)	0.001
*AM420042_s (Leptotrichia*)	0 (0, 0)	0.013 (0.214, 0)	0.002
***Centipeda periodontii***	0 (0.029, 0)	0.007 (0.443, 0)	0.036
***Selenomonas sputigenad***^d^	0 (0.037, 0)	0.022 (0.199, 0)	0.026

^a^Expressed as median and range.

^c^By Mann-Whitney U test.

^d^Species associated with gingivitis or periodontitis are bolded.
