# Supplementary material for: The presence of bacteria within tissue provides insights into the pathogenesis of oral lichen planus
Source: Sci Rep. 2016 Jul 7;6:29186. doi: 10.1038/srep29186 (PMC4935860; doi:10.1038/srep29186)
Supplement: Supplementary Information [file srep29186-s1.pdf]

The presence of bacteria within tissue provides insights into the pathogenesis of oral lichen planus

Yun Sik Choi, Yunji Kim, Hye-Jung Yoon*, Keum Jin Baek, Jehan Alam, Hee Kyung Park*, Youngnim Choi*

Supplementary materials and methods

Bacteria and human epithelial cell culture

S. sanguinis ATCC 804 (American Type Culture Collection, Manassas, VA, USA) and *S. gordonii* ATCC 10558 (ATCC) were cultured in brain heart infusion (BHI) medium at 37 °C under aerobic conditions. *C. gingivalis* KCOM 1581 (Korean Collection for Oral Microbiology, Gwangju, Korea) was cultured in BHI medium supplemented with 5 μg/ml hemin (Sigma, St Louis, MO, USA) plus 10 μg/ml vitamin K in an anaerobic atmosphere (5% H_2, 10% CO_2, and 85% N_2). Bacteria in the log phase were used for all experiments. For florescent application, bacteria were stained with 5 μM CFSE at room temperature for 20-30 minutes. Bacterial concentration was determined using the standard curves generated by OD at 600 nm and flow cytometric counts.

HOK-16B cells were maintained in keratinocyte growth medium with a supplementary growth factor bullet kit (Clonetics, Sandiego, CA, USA) in an atmosphere with 5% CO_2 at 37 °C.

Measurement of TER and cell viability after bacterial infection

HOK-16B cells (10^5 cells/well) were plated onto a 3 μm-pore-size polycarbonate filter of a 24-well plate of the transwell two-chamber tissue culture system. TER was assessed using an ERS Volt-Ohm Meter (Millipore Bedford, MA, USA). A resistance of the epithelial layer > 10 Ω is considered to imply a tight-junctioned epithelial layer. The cells were cultured for 2 or 3 days until a confluent monolayer reached the peak resistance of approximately 15 Ω. Then, the cells were infected with *S. sanguines*, *S. gordonii*, and *C. gingivalis* at the multiplicity of infection (MOI) of 500. TER was measured at 0, 6, 12, and 24 hours.

For the cell viability assay, HOK-16B cells (10^5 cells well^{-1} in 96-well plates) were cultured to a confluent monolayer and infected with *S. sanguines*, *S. gordonii*, and *C. gingivalis*

at MOI 500 for 24 hours. Next, 20 µl of CCK-8 solution was applied to each well, and the cells were further incubated for 1 hour at 37 °C. The absorbance was measured at 450 nm using a microplate reader. Cell viability was calculated as a relative percentage relative to the vehicle control.

Bacterial internalization into human cells

HOK-16B cells were plated at a density of 3 x 10^4 cells cm^{-2} onto 24-mm diameter glass cover slips. The cells were infected at 70% confluence with the CFSE-labeled bacteria at an MOI of 1,000 for 24 hours. Purified human $CD4^+$, $CD8^+$, or $CD14^+$ cells (2.5 x 10^5 cells) in RPMI medium with 10% fetal bovine serum (FBS) were infected with the CFSE-labeled bacteria at an MOI of 1,000 for 1 hour in the absence of antibiotics.

For confocal microscopic examination, the infected cells were fixed, permeabilized, and then stained with rhodamine-phallodin (Molecular Probes) and Hochest 33342 (Molecular Probes). The leukocytes were attached onto collagen-coated slides after staining. Mounted slides were imaged using a Zeiss LSM700 laser scanning confocal microscope (Carl Zeiss, Oberkochen, Germany) with serial z-sections.

For flow cytometric analysis, the infected cells were washed with PBS and resuspended in trypan blue (400 mg ml^{-1} prepared in 0.85% saline solution) to quench the fluorescence of the bacteria bound on the surface. The cells were analyzed using a FACSCalibur (BD Biosciences). The cells were gated first on the appropriate population based on the forward vs. side scatters. Then, live cells were gated based on the FL-3 fluorescence of trypan blue. Non-infected live cells and the cells fixed with 3.7% formaldehyde and infected with the same MOI of CFSE-labeled bacteria served as negative controls that were subtracted from the values by live cells.

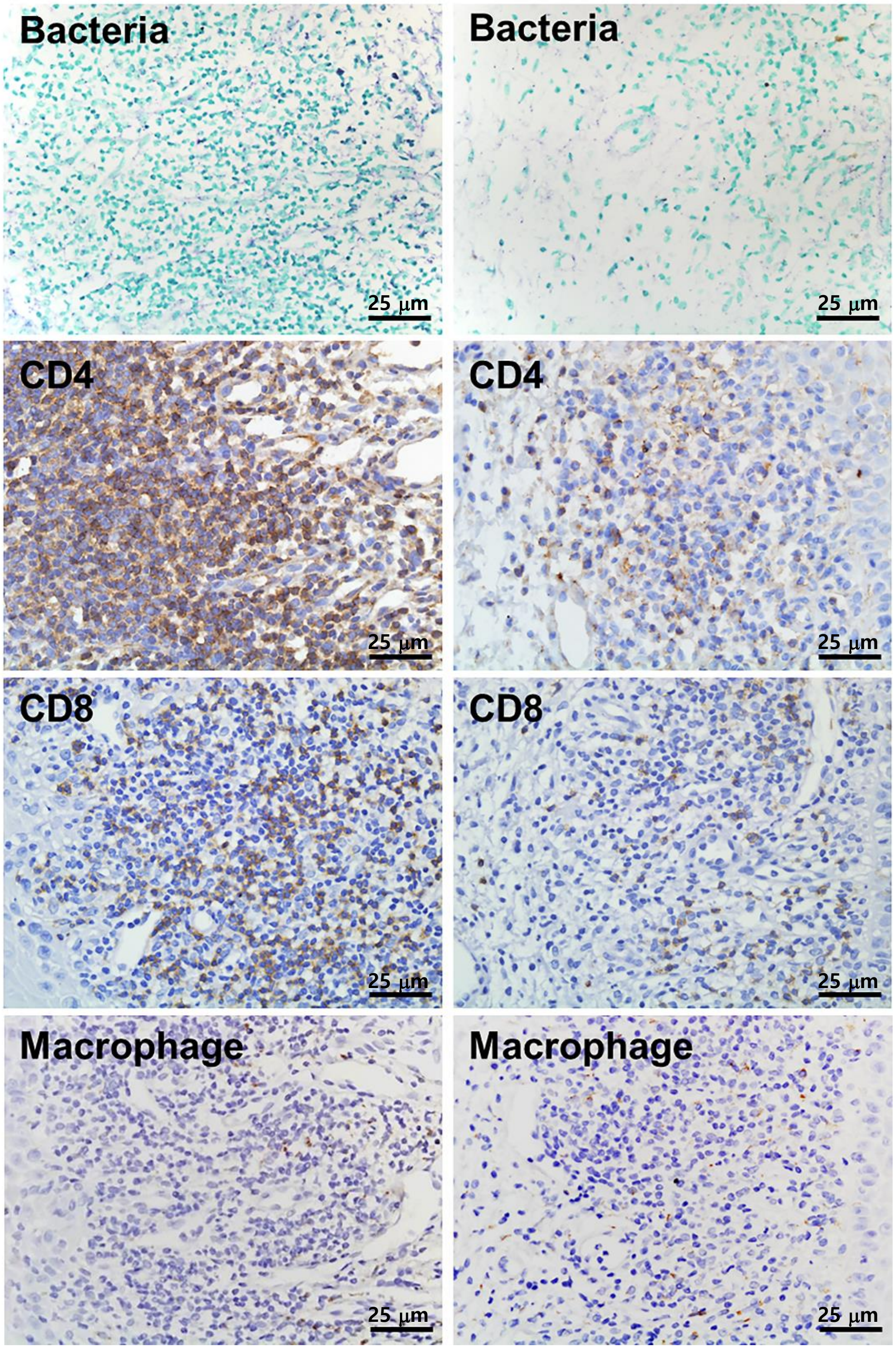

Supplementary Figure 1. The levels of bacteria, CD4$^+$ cells, CD8$^+$ cells, and macrophages in the areas with different degrees of inflammatory infiltration. Serial sections of OLP tissues were subjected to *in situ* hybridization with a eubacterial 16S rRNA probe and immunohistochemical staining of CD4, CD8, and macrophage. By comparison of each slide with H&E stained section, the same locations with heavy (left panels) or low (right panels) inflammatory infiltration were chosen and photographed.

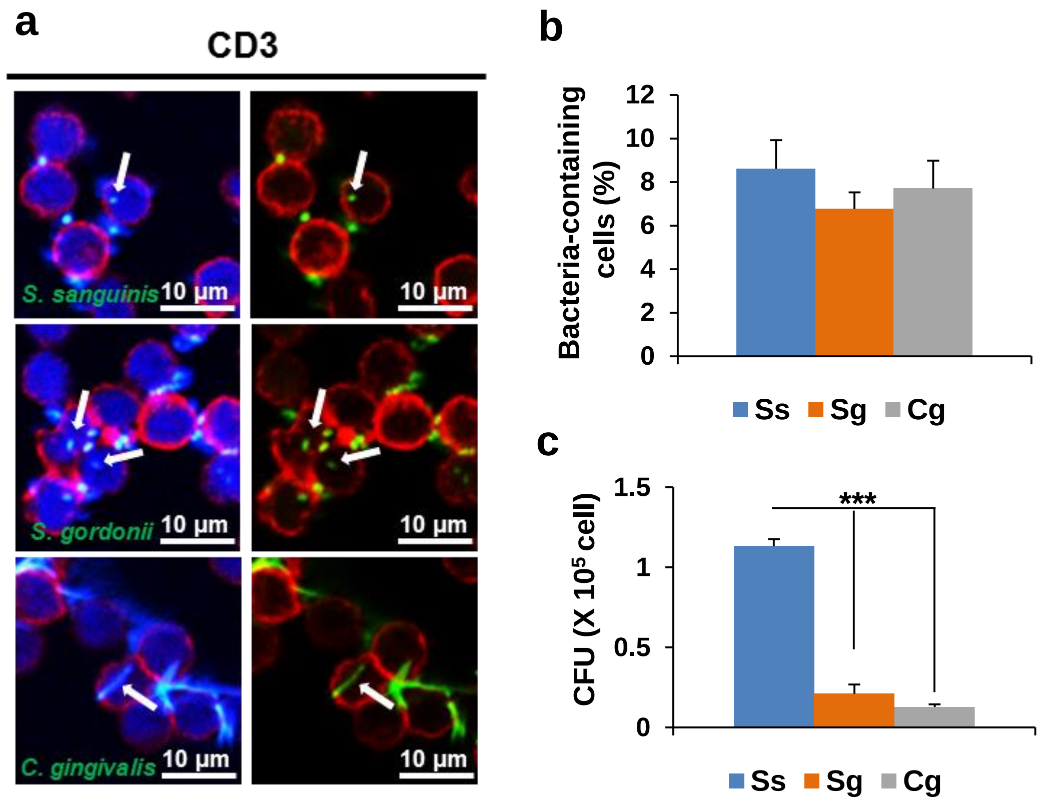

Supplementary Figure 2. Internalization of selected oral bacterial species into CD3⁺ T cells. Purified human CD3⁺ cells were infected with CFSE-labeled bacteria at MOI 1000 for 1 hour (n = 3 using cells from one donor). (a) Representative confocal microscopy of CFSE-labeled bacteria (green) internalized into human CD3⁺ cells. White arrows indicate bacteria within the cell boundary. Blue, Hoechst 33342; red, rhodamine phalloidin. (b) The percentage of cells containing the internalized bacteria analyzed by flow cytometry (s.e.m.). (c) Purified human CD3⁺ cells were infected with bacteria at MOI 1000 for 1 hour and further cultured for 1 hour in the presence of gentamicin. After lysing the cells, bacteria in the lysates were cultured on blood agar plates (***, $P < 0.001$ by one-way ANOVA with Tukey's post hoc).

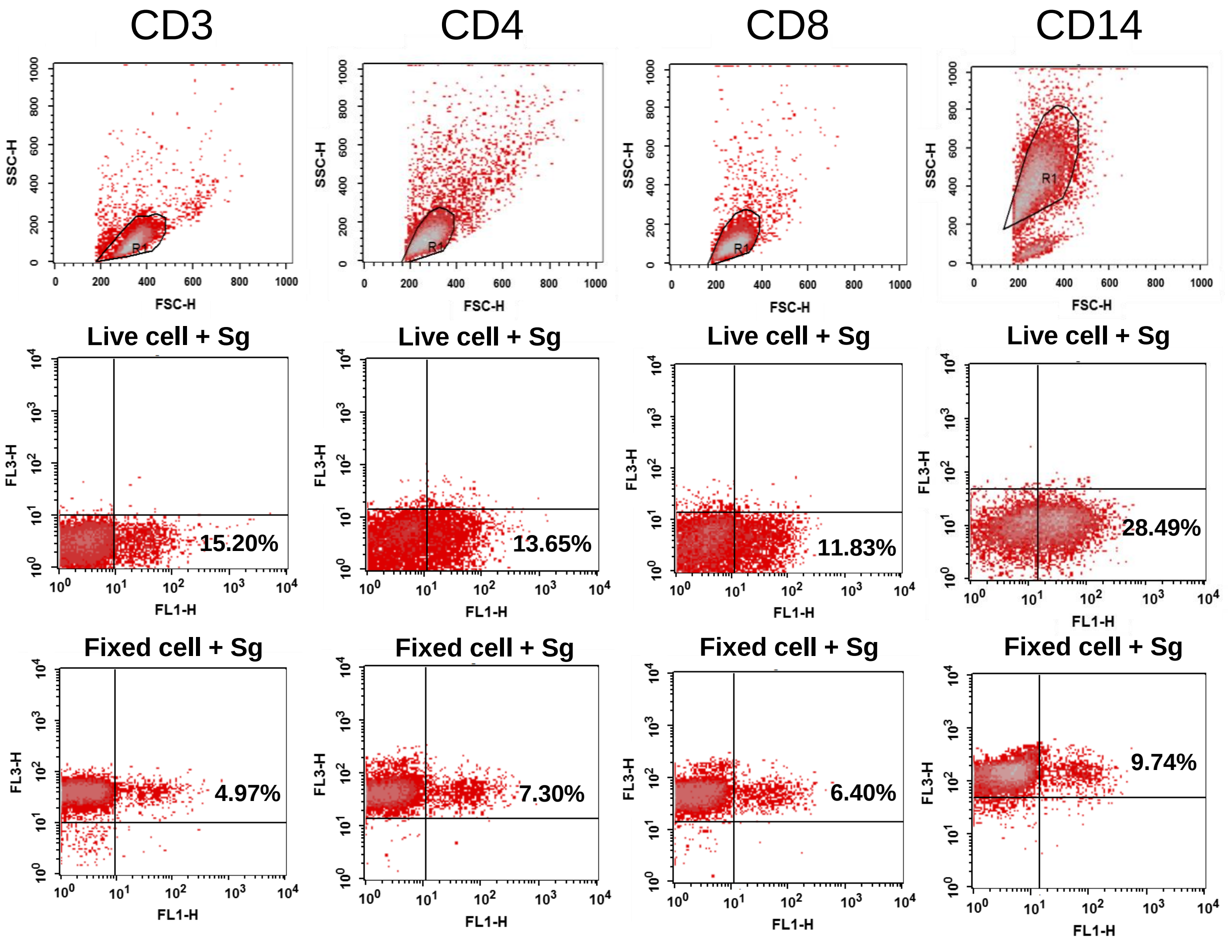

Supplementary Figure 3. Flow cytometric analysis of bacterial internalization into purified $CD3^+$, $CD4^+$, $CD8^+$, or $CD14^+$ cells. The cells were gated first on the appropriate population based on the forward vs. side scatters (Top panels). Then, cells were gated based on the FL-3 fluorescence of trypan blue (Middle panels). Bacterial internalization into host cells was calculated by subtracting the percentage of unquenched cells ($FL\text{-}1^+$) in the negative control cells (Bottom panels), which were fixed with 3.7% formaldehyde and infected with the same MOI of CFSE-labeled bacteria, from the percentage of $FL\text{-}1^+$ cells among the live cell population (Middle panels).

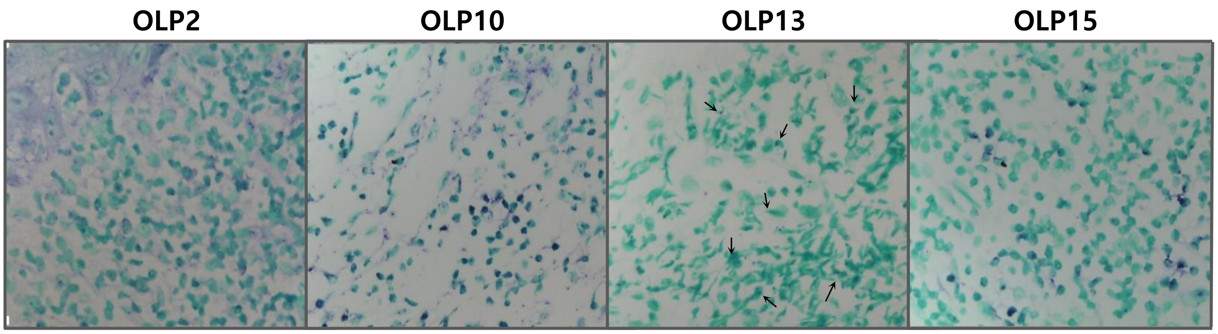

Supplementary Figure 4. *In situ* detection of bacteria in the laminar propria of OLP tissues from the patients who had no ulceration and limited erosive lesions. Arrows in OLP13 indicate bacterial signals overlapped with the nuclei.

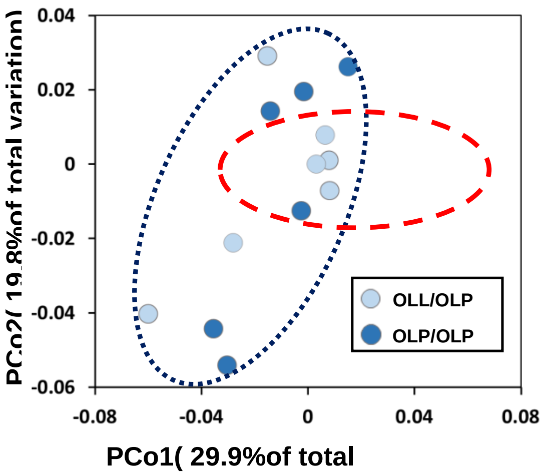

Supplementary Figure 5. PCoA plot of OLP/OLP and OLL/OLL cases. Red dotted line represents distribution of control samples

Supplementary Table 1. The detailed histopathological features of all 36 OLP cases

	1	2	3	4	5	6	7	8	9	10	11	12	13	14	15	16	17	18	19	20	21	22	23	24	25	26	27	28	29	30	31	32	33	34	35	36	37	Sum
OLP2	0	1	1	1	0	0	0	0	0	0	1	0	0	0	1	0	1	1	0	1	0	1	1	-1	-1	0	-1	1	-1	0	0	1	0	0	0	1	1	10
OLP3	0	1	1	0	0	0	0	0	0	0	1	0	0	0	1	0	1	1	0	1	0	0	1	0	0	1	0	1	-1	-1	0	1	1	0	0	1	1	12
OLP5	0	1	0	1	0	0	0	0	0	1	1	0	0	0	0	0	1	0	0	0	-1	1	1	-1	-1	0	-1	1	-1	-1	0	0	0	0	0	1	1	4
OLP6	0	1	0	0	1	0	0	1	0	0	1	0	1	0	0	0	1	0	1	0	0	0	1	0	0	1	0	1	-1	0	0	0	0	0	1	0	1	11
OLP8	0	1	0	0	1	0	0	1	0	0	1	0	0	0	0	0	1	1	0	1	0	0	1	-1	0	0	-1	1	-1	0	0	1	0	0	1	1	1	10
OLP9	0	1	0	1	0	0	1	0	0	0	1	0	0	0	0	0	1	1	0	1	0	0	1	-1	-1	0	-1	1	-1	0	0	0	0	0	1	1	0	7
OLP10	0	1	0	0	1	0	0	1	0	1	1	0	0	0	0	0	1	1	0	1	0	0	1	0	0	1	0	1	-1	-1	-1	0	0	1	1	1	1	12
OLP12	0	1	0	0	1	0	0	1	0	0	1	0	1	1	0	0	1	0	1	0	0	0	1	0	0	1	0	1	0	-1	0	1	0	1	0	1	1	14
OLP13	0	1	0	0	1	0	0	0	0	1	1	0	0	0	0	0	0	1	0	1	0	1	1	-1	-1	0	-1	1	-1	0	0	0	0	1	1	1	1	9
OLP14	0	1	0	0	0	1	0	1	0	0	1	0	0	1	0	0	1	0	1	0	0	0	1	0	0	1	0	1	0	0	-1	1	0	0	1	1	1	13
OLP15	0	1	0	0	1	0	0	1	0	0	1	0	0	0	0	0	1	0	1	0	0	0	1	0	0	1	0	1	-1	0	-1	0	0	1	1	1	0	10
OLP16	0	1	0	1	0	0	1	0	0	0	1	0	0	0	0	0	1	0	0	0	-1	0	1	-1	-1	0	-1	1	-1	0	-1	1	0	1	1	0	0	4
OLP19	0	1	0	0	1	0	0	1	0	0	1	0	0	0	0	0	0	0	1	0	0	0	1	0	0	1	0	1	0	0	0	1	0	1	0	1	0	11
OLP20	1	1	0	1	0	0	0	1	1	0	1	0	1	0	0	0	0	1	0	1	0	0	1	0	0	1	0	1	0	0	0	1	1	0	0	1	0	15
OLP21	0	1	0	0	0	0	0	1	0	0	1	1	0	0	0	0	1	0	1	0	0	0	1	0	0	1	0	1	0	0	0		0	1	0	1	0	11
OLP22	0	1	0	0	0	1	0	1	0	0	1	0	1	0	1	0	0	0	1	0	0	0	1	0	0	1	0	1	0	0	0	1	0	0	0	0	0	11
OLP23	0	1	1	1	0	1	0	1	0	0	1	0	0	0	0	0	1	0	1	0	0	0	1	0	0	1	0	1	-1	0	-1	0	0	0	0	0	0	9
OLP24	0	1	0	1	0	0	1	0	0	0	1	0	1	0	0	0	0	0	1	0	0	0	1	0	0	1	0	1	0	0	0	1	0	1	0	0	0	11
OLP25	0	1	0	1	0	0	0	1	0	0	1	0	0	0	0	0	0	0	1	0	0	0	1	0	0	1	0	1	0	0	0	0	0	1	0	0	0	9
OLP26	0	1	1	0	0	0	0	0	0	0	1	1	0	0	0	0	0	0	1	0	0	0	1	0	0	1	0	1	0	0	0	1	0	0	0	1	1	11
OLP27	0	1	0	1	0	0	0	1	0	0	1	1	1	0	0	0	1	0	1	0	0	0	1	0	0	1	0	1	0	0	0	0	0	1	0	1	1	14
OLP28	0	1	0	1	0	0	0	1	0	0	1	0	0	0	0	0	1	0	1	0	0	0	1	0	0	1	0	1	0	0	0	0	0	0	0	1	1	11
OLP29	0	1	0	1	0	0	0	1	1	0	1	0	0	0	0	0	0	0	1	0	0	0	1	0	0	1	0	1	0	0	0	1	0	0	0	0	1	11
OLP30	0	1	0	1	0	1	0	1	1	0	1	0	1	0	0	0	0	1	0	1	0	0	1	0	0	1	0	1	0	0	0	1	0	0	0	0	0	13
OLP31	0	1	0	1	0	0	0	1	0	0	1	0	1	0	0	0	0	0	1	0	0	0	1	0	0	1	0	1	0	0	0	1	0	1	0	0	1	12
OLP32	0	1	0	0	0	1	1	1	0	0	1	0	0	0	0	0	0	0	1	0	0	0	1	0	0	1	0	1	0	0	0	1	0	1	0	0	0	11
OLP33	0	1	0	0	0	1	0	1	0	0	1	0	0	0	0	0	0	0	1	0	0	0	1	0	0	1	0	1	0	0	0	1	0	1	0	0	1	11
OLP34	0	1	0	0	1	0	0	1	0	0	1	0	0	0	0	0	0	0	1	0	0	0	1	0	0	1	0	1	0	0	0	1	0	1	0	0	1	11
OLP35	0	1	1	0	0	1	0	0	0	0	1	0	1	0	0	0	0	0	1	0	0	0	1	0	0	1	0	1	0	0	0	1	1	1	0	0	0	12
OLP36	0	1	0	1	0	0	0	1	0	0	1	0	0	0	0	0	1	0	1	0	0	0	1	0	0	1	0	1	-1	0	0	1	0	1	0	0	1	11
OLP37	0	1	1	0	0	0	0	0	0	0	1	1	0	0	0	0	1	0	1	0	0	0	1	0	0	1	0	1	0	0	0	1	0	0	0	0	1	11
OLP38	0	1	1	0	0	0	0	0	0	0	1	0	0	0	0	0	1	0	1	0	0	0	1	0	0	1	0	1	0	0	0	1	1	1	0	0	1	12
OLP39	0	1	0	1	0	0	0	1	0	0	1	0	0	0	0	0	1	0	1	0	0	0	1	0	0	1	0	1	0	0	0	1	0	1	0	0	1	12
OLP40	0	1	0	0	0	1	0	0	0	0	1	0	0	0	0	0	1	0	1	0	0	0	1	0	0	1	0	1	0	0	0	1	0	1	0	0	1	11
OLP41	0	1	0	0	0	1	0	1	0	0	1	0	1	0	0	0	1	0	1	0	0	1	1	0	0	1	0	1	0	0	0	1	0	0	0	0	1	13
OLP42	0	1	1	0	0	0	0	0	0	0	1	1	0	0	0	0	0	0	1	0	0	0	1	0	0	1	0	1	0	0	0	1	0	0	0	0	0	9

[1]Hyperorthokeratosis in the epithelium

[2]Hyperparakeratosis in the epithelium

[3]Atrophy (reduction in thickness more than 1/3 of normal area) in the epithelium

[4]Acanthosis (broadening of rete ridges more than two time normal width for area) in the epithelium

[5]Simple hyperplasia (thickness more than 1 1/2 time normal thickness for area, excluding stratum corneum) in the epithelium

[6]Atrophy alternating with hyperplasia in the epithelium

[7]Finger-like rete ridges

[8]Sawtooth rete ridges

[9]Thick stratum granulosum more than five cell layers thick in the epithelium

[10]Easily visible small groups or heavy infiltration by leucocytes in the epithelium

[11]Liquefaction degeneration of basal layer of the epithelium

[12]Intraepithelial vesicles: subepithelial vesicles excluded epithelial cellular changes

[13]Colloid bodies – civatte bodies in the epithelium

[14]Multinucleated epithelial cells: cells containing three or more nuclei

[15]Hyperchromatism in the epithelium
[16]Epithelial dysplasia, slight, moderate or severe
[17]Supra-basilar apoptosis
[18]Band-shaped superficial inflammatory infiltrate, some areas
[19]Band-shaped superficial inflammatory infiltrate, all areas
[20]Not band-shaped superficial inflammatory infiltrate, some areas
[21]Not band-shaped superficial inflammatory infiltrate, all areas
[22]Superficial inflammatory infiltrate, focal/perivascular
[23]Intensity of superficial inflammatory infiltrate, moderate or heavy
[24]Deep inflammatory infiltrate located deep to superficial infiltrate, some or all areas
[25]Deep inflammatory infiltrate, focal/perivascular
[26]Intensity of deep inflammatory infiltrate: none
[27]Intensity of deep inflammatory infiltrate: slight
[28]Cell types of inflammatory infiltrate, lymphocytes and histiocytes in the
[29]Cell types of inflammatory infiltrate, plasma cells
[30]Cell types of inflammatory infiltrate, neutrophils
[31]Cell types of inflammatory infiltrate, eosinophils
[32]Juxtaepithelial cell-free zone
[33]Hyalinization of collagen in juxtaepithelial area
[34]Melanophages in juxtaepithelial area
[35]Oedema in juxtaepithelial area
[36]Dilatation of vessels in the connective tissue
[37]Neutrophils in lumen of vessels in the connective tissue
1, positive features; -1, negative features
, cases diagnosed as OLL by one pathologist
, cases diagnosed as OLL by two pathologists

Supplementary Table 2. Species/phylotypes decreased or increased in the subset of OLP samples that presented complete separation from the control samples in PCoA plot

	Decreased compared to control	Increased compared to control
Species/phlyotype common to Table 2	*Escherichia coli group*	*Fusobacterium nucleatum*
	Streptococcus vestibularis	*Neisseria oralis*
	Streptococcaceae_uc_s	*Capnocytophaga gingivalis*
	EF016847_s (EF016847_g)	*Leptotrichia hongkongensis*
		Prevotella oulorum
		AF385572_s (*Leptotrichia*)
		Treponema denticola[d]
		Treponema socranskii[d]
		AF385554_s (*Prevotella*)
		Centipeda periodontii
Additional species/phlyotype	*Streptococcus pseudopneumoniae*	*Capnocytophaga granulosa*
	Streptococcus mitis	*Fusobacterium canifelinum*
	Gemella haemolysans	*DQ012324_s* (*Capnocytophaga*)
	Streptococcus oralis	*Lachnoanaerobaculum saburreum*
	HQ757980_s (*Streptococcus*)	*Actinomyces dentalis*
		AM420230_s (*Tannerella*)
		Leptotrichia hofstadii
		JN713365_s (*Treponema*)
		Actinomyces_uc
		ADCM_s (Paludibacter)
		Selenomonas_g1_uc
		Selenomonas infelix
		4P005522_s (*Actinomyces*)
		Prevotella maculosa
		Leptotrichia_uc
		Fretibacterium fastidiosum
		FJ976257_s (TM7)
		Prevotella buccae
		4P003105_s (*Selenomonas_g1*)
		EU592964_s (*Olsenella*)
		Prevotella veroralis
		AF385503_s (*Selenomonas_g1*)
		Actinomycetaceae_uc_s
		4P003904_s (*Fusobacterium*)
		4P003388_s (*Selenomonas_g1*)
		Treponema_uc

Supplementary Table 3. Relative abundance (%)[a] and distribution of the 31 species/phylotypes in OLL/OLP and OLP/OLP groups

Species/phylotypes	Control (n = 11)	OLL/OLP (n = 7)	OLP/OLP (n = 6)	P^b	P^c
HQ767899_s (*Streptococcus*)	2.401	0.007	0.657	**0.010**	0.108
Escherichia coli group	0.449	0.083	0.046	**0.035**	**0.031**
Fusobacterium nucleatum	0.191	1.196	1.215	0.076	0.075
Neisseria oralis	0	0.068	0.168	**0.022**	**0.008**
Capnocytophaga gingivalis	0.089	0.213	0.090	**0.013**	0.725
Leptotrichia hongkongensis	0.020	0.371	0.042	**0.004**	0.404
Stomatobaculum longum	0.025	0.079	0.296	0.090	**0.036**
Aggregatibacter segnis	0	0.070	0.065	0.052	**0.049**
Actinomyces meyeri	0.018	0.089	0.532	0.063	**0.010**
Eikenella corrodens	0.014	0.065	0.055	**0.014**	**0.049**
4P004975_s	0.039	0.009	0	0.143	**0.006**
EF016847_s (EF016847_g)	0.065	0.008	0	**0.014**	**0.014**
Capnocytophaga sputigena	0.025	0.205	0.146	**0.006**	**0.008**
Leptotrichia buccalis	0	0.010	0.079	0.123	**0.004**
Prevotella oulorum	0	0.018	0.033	0.076	0.075
Streptococcus vestibularis	0.033	0	0	**0.002**	**0.002**
Megasphaera micronuciformis	0	0.026	0.066	0.052	**0.001**
AF385572_s (*Leptotrichia*)	0	0.073	0.020	**0.006**	**0.043**
AF385518_s	0	0.010	0.050	0.31	**0.037**
Myxococcus virescens group	0	0.047	0	**0.002**	0.176
Mogibacterium vescum	0	0.008	0.072	0.240	**0.008**
Blautia wexlerae	0	0.032	0	**0.002**	**0.048**
AF385506_s (TM 7_f)	0	0.029	0.055	0.123	**0.024**
Treponema denticola	0	0.024	0.028	**0.047**	**0.039**
Treponema socranskii	0	0.012	0.042	0.143	**0.049**
AF385554_s (*Prevotella*)	0	0.010	0.036	0.123	**0.019**
AY134896_s (*Leptotrichia*)	0	0.007	0	**0.006**	**0.048**
Streptococcaceae_uc_s	0.023	0	0	**< 0.001**	**0.039**
AM420042_s (*Leptotrichia*)	0	0.007	0.018	**0.006**	**0.001**
Centipeda periodontii	0	0	0.018	0.12	**0.018**
Selenomonas sputigena	0	0.007	0.022	0.076	**0.010**

[a]median

[b]Control vs. OLL/OLP by Mann-Whitney U test, boldness for significant values

[c]Control vs. OLP/OLP by Mann-Whitney U test, boldness for significant values
